# Trochanteric osteotomy versus posterolateral approach: function the first year post surgery. A pilot study

**DOI:** 10.1186/1471-2474-12-138

**Published:** 2011-06-26

**Authors:** Margot van der Grinten, Max Reijman, Frans C van Biezen, Jan AN Verhaar

**Affiliations:** 1 Dept. of Orthopaedic Surgery, Erasmus MC, P.O. Box 2040, 3000 CA Rotterdam, The Netherlands

## Abstract

**Background:**

Although no prospective studies have compared functional results of trochanteric osteotomy and a non-trochanteric approach, most surgeons feel that trochanteric osteotomy is outdated in simple hip arthroplasty. Reasons not to perform an osteotomy include the fear of longer rehabilitation and worse (final) functional outcome.

**Method:**

This prospective study examines differences in rehabilitation between posterolateral and trochanteric approach one year post-surgery using questionnaires (WOMAC, SF-36, HHS) and functional tests (walking, climbing stairs, rising from sitting, and strength tests). Of the 109 patients 24 had a trochanteric osteotomy: the selected approach was based on the surgeon's preference. The trochanteric osteotomy group included more patients with developmental dysplasia of the hip. Before the start of the study no power analysis was performed.

**Results:**

Data from the questionnaires showed no significant differences between the two groups at 3, 6 and 12-months follow-up. At 3-months follow-up patients in the trochanteric osteotomy group scored lower on the functional tests. This difference had disappeared at 6 and 12-months follow-up, except for abduction force which remained lower in the trochanteric osteotomy group in patients with a non union of the TO.

**Conclusion:**

For simple hip arthroplasty an approach without osteotomy seems a logical choice. Although the power of this study is low, in experienced hands trochanteric osteotomy seems to give good functional results at 6-12 months post surgery if trochanteric union is obtained. Therefore, one should not hesitate to perform an osteotomy in difficult cases.

## Background

Nowadays most surgeons feel that trochanteric osteotomy (TO) is obsolete in primary hip surgery and the technique seems to be falling into disuse. In revision arthroplasty extended TO is increasingly used. In the past functional results after TO have been ambiguous.

Total hip arthroplasty (THA) without osteotomy of the greater trochanter is reported to have a shorter operating time (30-60 min), less blood loss (1-2 units of blood), no trochanteric problems (e.g. trochanteric bursitis, non-union) and a faster rehabilitation compared to THA with osteotomy [1-3]. Although these procedures are well documented, no prospective studies compared (final) functional outcome after THA with and without TO. Some even describe a lower revision rate after TO with Charnley prostheses [4]; whereas others conclude that abduction strength is lower when *no *osteotomy is performed [5,6] and that it is not a functional problem when there is no union of the trochanter in revision surgery [5,6]. Also, in complex cases, more precise positioning of the hip prosthesis is possible after TO [2,7]. Nevertheless, TO for THA is nowadays rarely performed in simple primary procedures, mainly due to fear of worse functional outcome. Although rehabilitation seems to be faster without TO, to our knowledge no prospective studies have compared functional outcome of the two techniques.

In our clinic both the TO and the posterolateral (PL) approach are used by orthopaedic surgeons for patients with primary THA. Therefore, the present study compares both techniques in patients with a primary THA with regards to functional outcome at 1 year post-surgery.

## Methods

All consecutive patients receiving a primary THA between September 2003 and February 2005 (n = 173) were eligible for inclusion. Excluded were patients with insufficient command of the Dutch language (spoken and/or written), patients unable to mobilise unrestrictedly due to other causes than for which surgery was planned, and patients planned for THA after arthrodesis or girdlestone of the hip.

Finally, 109 patients were included. Of these, in 24 patients the TO was performed or supervised by one member of the staff; this surgeon preferred the TO approach of the hip. Generally, the approach was selected by the surgeon who was performing the operation.

TO was performed as described by Charnley [8]. The trochanter was fixed with 2 double wires. After TO patients were allowed partial (10%) weight bearing for 6 weeks in order to allow trochanteric union.

The PL approach was performed or supervised by four members of the orthopaedic staff. In these cases the patients were positioned in a stable lateral position. The short external rotators were incised to gain access to the hip joint. After implanting of the hip, the posterior capsule was closed and the external rotators fixed to the trochanter. After the PL approach patients were allowed full weight bearing.

In both groups pre-operative templating was performed to recreate the anatomical situation as accurately as possible.

Information on age, weight and height for calculation of body mass index (BMI), American Society of Anaesthesiologists (ASA) score, Kellgren & Lawrence (K&L) score and indication for surgery was collected. Preoperatively all patients were asked to fill in the Western Ontario and McMaster Universities (WOMAC) Index of osteoarthritis and the Medical Outcomes Study 36-Item Short-Form Health Survey (SF-36). The following functional tests were performed; patients were asked to walk 50 meters, rise from a chair 3 times and ascend/descend 5 stairs. Time to perform each task was recorded. Hip abduction torque for both hips was measured with a handheld dynamometer with the patient lying on his/her side. The dynamometer was positioned on the lateral epicondyle of the distal femur. Maximum abduction force after three attempts was scored. Knee extension force was measured with the patient sitting down and the knee in 90° flexion. The Harris Hip Score (HHS) was acquired. One examiner (following a standard protocol) conducted all tests in all patients. Length of stay in the hospital was also noted. During follow-up at 3, 6 and 12 months the questionnaires were filled-in again, the functional tests were repeated, and any complications were noted. If a complication was scored, patients were still included in the study and the tests were performed if possible.

The local Medical Ethics Committee approved the study and written informed consent was obtained from each patient.

### Statistical analysis

First, it was established whether the variables had a normal distribution using the normality Shapiro-Wilk test. Based on these analyses, the results are presented as means and standard deviations (SD).

Differences between both techniques in patients with a primary THA with regards to functional outcome the first year post-surgery were evaluated using independent t-tests (for normally distributed variables) or by Pearson Chi-square test (for categorical variables).

Differences between abduction force in patients with a TO with or without consolidation was evaluated with the Mann-Whitney test.

Analyses were performed using SPSS 17.0 (SPSS Inc., Chicago, USA). An alpha value of 0.05 was set as the level of significance.

## Results

Table 1 presents the baseline characteristics of the study population. The indication for THA differed slightly between the two groups (Table 2). In the PL group more patients had primary osteoarthritis, whereas in the TO group more patients had hip dysplasia. Stay in hospital was longer in the TO group than in the PL group, i.e. 12.0 (range 5-31) days versus 8.2 (range 3-22) days, respectively, and fewer patients in the TO group were dismissed to their own home, i.e. 54.2% versus 83.5% respectively.

**Table 1:** Baseline characteristics of the study population

		Posterolateral		Trochanteric		No of patients	
Number of patients		85		24		109	

Male patients, number (%)		30	(35.3)	10	(41.7)	40	(36.7)

Age, mean (SD) in years		63.1	(15.2)	58.8	(15.4)	62.1	(15.2)

Body mass index, mean (SD) kg/ m^2^		26.8	(5.1)	25.0	(3.8)	26.4	(4.9)

Kellgren & Lawrence score: number (%)	1	3	(3.6)	2	(8.7)	5	(4.7)
	2	16	(19.3)	6	(26.1)	22	(20.8)
	3	40	(48.2)	8	(34.8)	48	(45.3)
	4	24	(28.9)	7	(30.4)	31	(29.2)

Left side, numer (%)		43	(50.6)	13	(54.2)	56	(51.4)

ASA score: number (%)	1	18	(21.2)	8	(33.3)	26	(23.9)
	2	45	(52.9)	12	(50.0)	57	(52.3)
	3	22	(25.9)	4	(16.7)	26	(23.9)

All p-values > 0.1

**Table 2:** Indications for joint replacement in the study population

	Posterolateral (n = 85) Number (%)		Trochanteric osteotomy (n = 24) Number (%)	
Primary osteoarthritis	61	(71.7)	13	(54.2)
Posttraumatic osteoarthritis	2	(2.4)	1	(4.2)
Dysplasia	7	(8.2)	4	(16.7)
Rheumatic disease	3	(3.5)		
Femoral neck fracture	2	(2.4)	2	(8.3)
Pseudarthrosis after fracture			2	(8.3)
Osteonecrosis	8	(9.4)	2	(8.3)
Perthes	2	(2.4)		

P-value = 0.121

At 3-months post-surgery 2 patients with a dislocation were no longer willing to participate, and 1 patient was lost to follow up. At 6 months, 2 patients were no longer willing to participate (1 dislocation), and at 12 months 6 patients (3 TO, 3 PL) were lost to follow-up.

**Figure 1:**
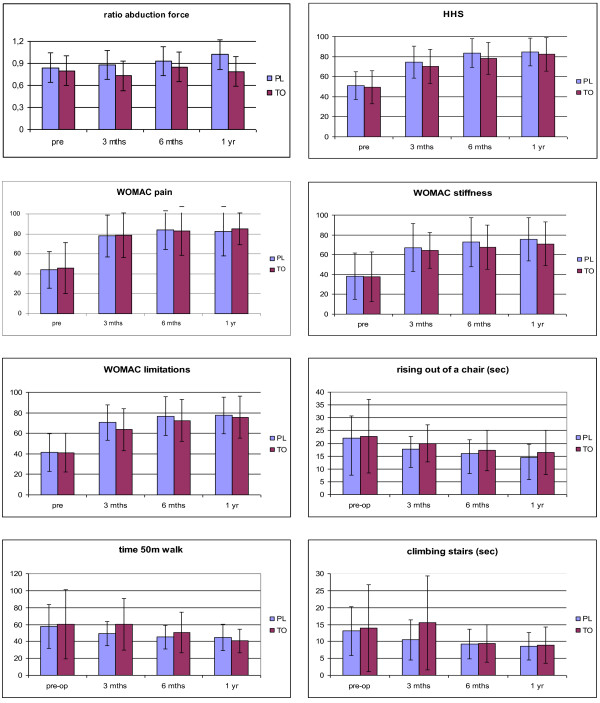
**Results of data from the questionnaires and functional tests in the posterolateral (PL) and the trochanteric osteotomy (TO) groups the first year post-surgery**.

No significant differences were found between the groups for the WOMAC or SF-36 pain scores (Figure 1), for the WOMAC limitations and SF-36 scales Physical functioning and Role physical, or for the HHS. For the other functional tests, at 3-months post-surgery the TO group had lower scores, except for the knee extension force which was similar in both groups. At 6-12 months post-surgery the between-group differences in function had disappeared, except for the abduction force which remained lower in the TO group (difference not significant).

### Complications

In the PL group, one patient had a haematoma. In the TO group one dislocation of the hip occurred within 3-months post-surgery; this patient had a pseudarthrosis of the trochanter, which was refixed because of repeated dislocation. Of the 24 trochanters, 4 did not consolidate. In 3 patients the trochanter was retracted over 2 cm. A difference in abduction force was noted between patients with and without consolidation of the trochanter (Table 3)

**Table 3:** Abduction force ratio after trochanter osteotomy

Trochanteric consolidation	Ratio abduction force post-operative/ pre-operative		
	
	3 months post surgery	6 months post surgery	12 months post surgery
YES(20)	0.87	1.03	1.12

NO (4)	0.49 p = 0.065	0.64 p = 0.026	0.55 p = 0.056

In the PL group one dislocation occurred within 2 weeks post-surgery; after several dislocations a revision was performed for a retroversed position of the cup after which the hip was stable. In addition, between 3 and 6 months post-surgery 2 patients in the PL group had a dislocation of the hip; both were treated with a restrained cup after several dislocations.

## Discussion

In the Netherlands, TO is rarely performed for primary THA. In more complex primary or revision cases, some surgeons perform an osteotomy for better exposure of the hip; i.e. the existing anatomy can be prepared more adequately and the components can be removed and placed more precisely [1,2]. However, even in these cases there is some reluctance to perform TO; this is probably due to fear of worse functional outcome and/or lack of experience with this procedure.

In previous studies the reported functional results of TO are ambiguous. In the present study, to provide better insight into functional results after TO, patients not only completed questionnaires but also performed functional and strength tests.

The present study shows that patients with a TO had a longer stay in hospital (12 vs. 8.2 days) and fewer releases to a home situation (54 vs. 83%). This difference between the groups may be because in the first 6 weeks after TO, patients were allowed only 10% weight bearing to enable trochanteric union. This may also explain the difference in functional tests up to 3 months post-surgery, which show a tendency for faster rehabilitation after the PL approach. However, these differences were not significant and had disappeared 6-12 months post-surgery, except for abduction force which remained lower in the TO group for the entire follow-up period.

If the TO did not consolidate the abduction force was lower than when consolidation did occur; this difference was only significant at 6 months post-surgery. Had there been a larger study population, this difference might also have been significant at 3 and 12 months post-surgery.

A limitation of the present study is that different surgeons operated on both groups, which might cause some bias related to the surgeon's expertise. However, based on the extensive experience of all participating surgeons in the surgical approach of their choice, we expect any influence to be minimal. Another limitation is that patients were not randomised over the two groups. Depending on which surgeon saw the patient at the outpatient clinic, they were operated with or without TO based on the surgeon's preference. Therefore, indications for THA differed slightly between the two groups. In the TO group slightly less patients had primary osteoarthritis and twice as many patients had a dysplastic hip compared with the PL group. Because surgery in these patients is often more complicated, rehabilitation may take somewhat longer. Had the two groups been more comparable at baseline, the results may have showed smaller differences. As only one surgeon performed the TO, this group was smaller than the PL group.

Before starting the study no power analysis was performed. All consecutive patients were included during the study period. An evaluation of functional outcome of both approaches was done 1 year after surgery. Because of the small difference between the groups and the relatively large standard deviation, the power of this study was low for the used clinical outcome. The number of patients needed to find a difference between both groups as reported in the current study (a non-inferiority study) with an alpha of 0.05 and a power of 0.8, are presented in table 4.

**Table 4:** Power analysis, and numbers needed for non-inferiority study

	Power current study	Numbers needed per approach (total) *	
HHS	21.1%	398	(796)

WOMAC pain	15.2%	469	(930)

WOMAC stiffness	33.4%	278	(556)

WOMAC function	10.7%	1197	(2394)

* numbers needed to perform a non-inferiority study, with α = 0.05 and power of 0.8 (1 - β), based on the results of the current study

Most surgeons have discarded TO as a surgical approach, based on previous studies showing a shorter operation time, less blood loss and faster rehabilitation without TO. Generally, rehabilitation was scored based on questionnaires, by performing strength tests or by examining clinical notes retrospectively [1,3,9].

Parker et al. compared duration of rehabilitation duration in 100 patients after TO and 100 patients in whom the trochanter was left intact [1]. Rehabilitation was evaluated as the number of days it took for patients to be able to sit, stand and walk. Data were obtained from notes made by the physiotherapist. Rehabilitation was faster (1 day) and patient admission time was slightly shorter (2-3 days on a total of 21 days) when the trochanter was left intact.

Wiesman et al. compared 12 patients with a THA on both sides; one side was with and the other side without TO [3]. They scored functional results based on clinical history notes, manually tested abduction force and by gait analysis. The authors reported a shorter operating time with less blood loss on the side without TO. Trochanteric problems were only seen on the side with TO. In gait analysis, abduction force measurement and HHS showed no significant differences. Although patients were satisfied with both sides, they had a slight preference for the side without TO.

## Conclusion

In simple primary or revision THA it seems logical to choose the approach with faster rehabilitation, less blood loss and faster, more frequent dismissals to home. As the power of this study is moderate, conlusions need to be drawn with caution. Nonetheless, in experienced hands TO seems to give good functional results 6-12 months after surgery if trochanteric union is obtained and therefore should not be discarded.

## Competing interests

The authors declare that they have no competing interests.

## Authors' contributions

MG contributed in the conception and design of the study, in the acquisition of data, analysis and interpretation of data, drafting the manuscript and final approval of the version to be published. MR was involved in the analysis and interpretation of data, revising of the manuscript and with the final approval of version to be published. FB contributed to the analysis and interpretation of data, revising manuscript and with the final approval of version to be published. JV	contributed to the conception and design of the study, analysis and interpretation of data, revising the manuscript and to the final approval of version to be published

## Pre-publication history

The pre-publication history for this paper can be accessed here:

http://www.biomedcentral.com/1471-2474/12/138/prepub
